# Histological Patterns of Epithelial Alterations in Keratoconus

**DOI:** 10.1155/2020/1468258

**Published:** 2020-07-30

**Authors:** Sara Crespo Millas, José Carlos López, Elena García-Lagarto, Estibaliz Obregón, Denise Hileeto, Miguel J. Maldonado, J. Carlos Pastor

**Affiliations:** ^1^Hospital Clínico Universitario de Valladolid (HCUV), Valladolid, Spain; ^2^Instituto Universitario de Oftalmobiología Aplicada (IOBA), Universidad de Valladolid, Valladolid, Spain; ^3^School of Optometry and Vision Science, University of Waterloo, Waterloo, Canada; ^4^Red Temática de Investigación Colaborativa en Oftalmología (OftaRed), Instituto de Salud Carlos III, Madrid, Spain

## Abstract

**Purpose:**

The purpose of this study was to confirm the presence of specific patterns of epithelial response in corneal buttons from keratoconus patients.

**Methods:**

This was a retrospective and descriptive study. 90 penetrating keratoplasty specimens obtained from patients diagnosed with keratoconus were evaluated using bright-field microscopy. Morphologically identifiable characteristics including epithelial cell density and epithelial thickness were analyzed on hematoxylin and eosin- (H&E-) and periodic acid of Schiff- (PAS-) stained slides.

**Results:**

Three distinctive patterns of epithelial alteration of the central cornea were established. Pattern 3, in which the central epithelium was as thick as peripheral epithelium, was the commonest (44.4%), followed by the pattern 2, defined as central epithelium thinner than periphery epithelium (38.9%), and the uncommonest pattern was number 1, with central epithelium thicker than the periphery (16.7%).

**Conclusions:**

Three distinctive histologic patterns that could potentially have a diagnostic and prognostic value in keratoconus patients were found.

## 1. Introduction

Keratoconus (KC) is a chronic progressive bilateral and asymmetric corneal ectasia affecting approximately 1/2,000 people in North America [1]. Nevertheless, recent studies have shown that this prevalence is much higher due to the improvement in corneal topography that allows KC diagnosis in earlier stages [2]. Histological and imaging parameters are currently used to guide the diagnostic and treatment approaches [3–5].

In KC, the central cornea becomes thinner and acquires a conical shape with a central protrusion. This leads to high levels of astigmatism associated with high amount of optical aberrations, monocular diplopia, abnormal image contrast, and associated dry eye symptoms.

The exact cause and underlying mechanisms of the disease are still not completely known, and several studies propose a multifactorial origin. One of the most frequent risk factors of progression is eye rubbing, commonly related with vernal conjunctivitis or allergic eye syndrome [6].

Clinical diagnosis is based on slit-lamp finding (corneal thinning, Vogt's striae, or Fleischer ring) and corneal topography; although in recent years, some work has been performed to identify biomarkers, mainly in tear fluid. The expanding knowledge of the tear proteome, lipidome, and metabolome has opened new ways to study KC and to identify possible diagnostic and/or prognostic biomarkers for this condition [7].

Also, in recent years, new imaging techniques based on optical coherence tomography (OCT) have demonstrated their usefulness and reliability as noninvasive methods. Even more, OCT has detected alterations in corneal epithelial thickness and distribution in KC, and it has a role in assessing Descemet's membrane detachment in acute corneal hydrops (ACH) and the depth of the demarcation line following corneal collagen cross-linking [8].

With the aid of high-resolution ultrasound digital scan, Reinstein et al. have identified the compensating role of the epithelium in very early stages of KC. This finding could allow to diagnose KC in patients who have a corneal topography “within normal limits” [9, 10].

KC patients are initially treated by conservative means with different types of contact lenses, but comfort is difficult to achieve in long-term due to the multifactorial nature of the disorder and the high rate of complications and treatment failure associated with the unpredictable rates of progression, which prevent adequate vision correction [11, 12]. As the condition progresses, more invasive treatments, including corneal cross-linking (CXL), corneal rings, and lamellar or penetrating keratoplasty, are needed [13].

Histopathological changes in KC include progressive corneal thinning and irregularity, Bowman membrane breaks, increased stromal density, spatial disorganization, and epithelial abnormalities [14, 15].

Thinning of the corneal stroma is an established fact in KC [16]. However, significant differences and conflicting evidence exist in terms of histopathological characteristics of the corneal epithelium in KC and their impact on treatment approaches and rates of complications.

Some studies involving histological analysis of corneas with KC demonstrate significant thinning of the central epithelium [17]. Nevertheless, other studies reported thickened epithelium in KC [18–20] or no differences in epithelial thickness between KC and normal controls [21].

Analysis of corneal buttons obtained after penetrating keratoplasty in our laboratory revealed the existence of three patterns in the corneal epithelium of patients with KC, which may have an important role in a more accurate interpretation of the new imaging techniques used for in vivo early diagnosis of the disease [22].

The purpose of this paper has been to describe the different epithelial patterns found in a relatively large series of penetrating keratoplasties in patients diagnosed of KC.

This type of study has not been performed so far and has the added value of having it done on penetrating keratoplasty buttons, which due to the increasingly widespread use of lamellar keratoplasties, is a difficult material to obtain nowadays and should prove superior for evaluating the epithelium because it involves less surgical manipulation than lamellar buttons.

## 2. Methods, Intervention, or Testing

This is a retrospective and descriptive study performed in accordance with the tenets of the Declaration of Helsinki, and all procedures were approved by the University of Valladolid ethics committee. Informed consent was obtained from all patients as a routine procedure before surgery.

Ninety penetrating keratoplasty specimens were selected from the archives of IOBA Ocular Pathology Laboratory and Hospital Clínico Universitario de Valladolid from those processed between 1986 and 2018. Initially, some samples were discarded because they showed artifacts as stromal scar or epithelial alterations due to processing of the samples and did not allow a correct measurement of the epithelial layer in all the zones. All paraffin blocks were retrieved, and 5 *µ*m-thick sections were obtained using a microtome (Leica Biosystems, Nussloch, Germany) and stained with hematoxylin and eosin (H&E) and periodic acid of Schiff (PAS). The histopathological slides were evaluated using bright-field microscopy (Leica DMLB4000, Leica Microsystems, Wetzlar, Germany).

Morphologically identifiable characteristics including epithelial cell density and epithelial thickness were analyzed.

Central and peripheral corneal epithelial thicknesses and total corneal thickness were also measured using Leica Application Suite software (Leica Microsystems, Wetzlar, Germany) with the caliper option. Diameter of the corneal button was always 8 mm. “Periphery” was defined as the outer 2 mm ring-shaped area and “center” as an interval of 4 mm in the central section. Three measurements of each sample in H&E and PAS were obtained in the center of the corneal button and in the periphery, and the mean value was calculated. The measurements of the epithelium thickness were obtained from the basal membrane to the more superficial epithelial layer, and the measurements of the total corneal thickness were obtained from the endothelium to the more superficial epithelial layer. The histopathological slides were evaluated by three of the authors (SCM, EGL, and EO).

Descriptive statistics are provided as the average and standard deviation in normally distributed data and the median and interquartile range in nonnormally distributed data. To explore for differences in epithelial thickness among groups, the one-way analysis of variance test was used for normally distributed data. All statistical tests were two-tailed; *p* < 0.05 was considered statistically significant.

## 3. Results

**Figure 1:**
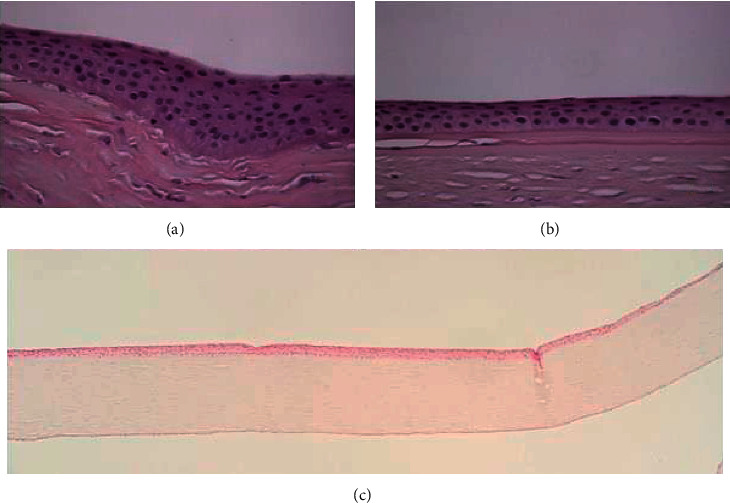
Pattern 1: increased corneal epithelial thickness with focal hyperplasia in the central cornea (a) and normal thickness in the peripheral cornea (b). H&E-stained section. Original magnification 40x (a and b) and 5x (c). H&E: hematoxylin and eosin.

**Figure 2:**
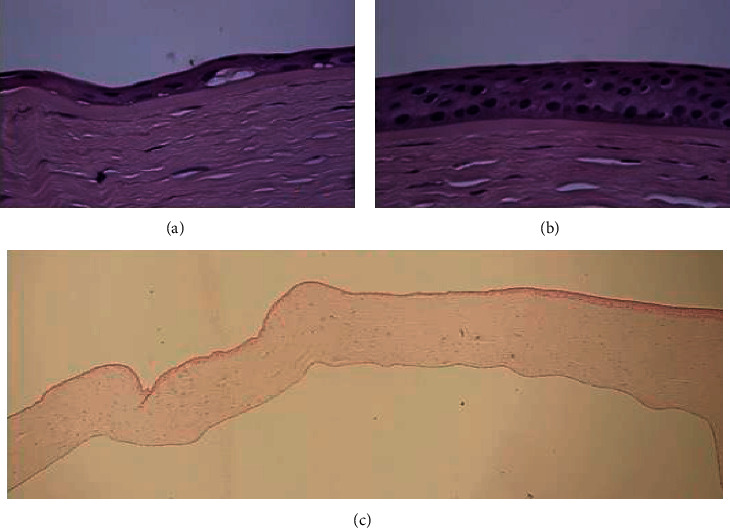
Pattern 2: markedly reduced central epithelial thickness with atrophy (a) and normal thickness in the peripheral cornea (b). H&E-stained section. Original magnification 40x (a and b) and 5x (c). H&E: hematoxylin and eosin.

**Figure 3:**
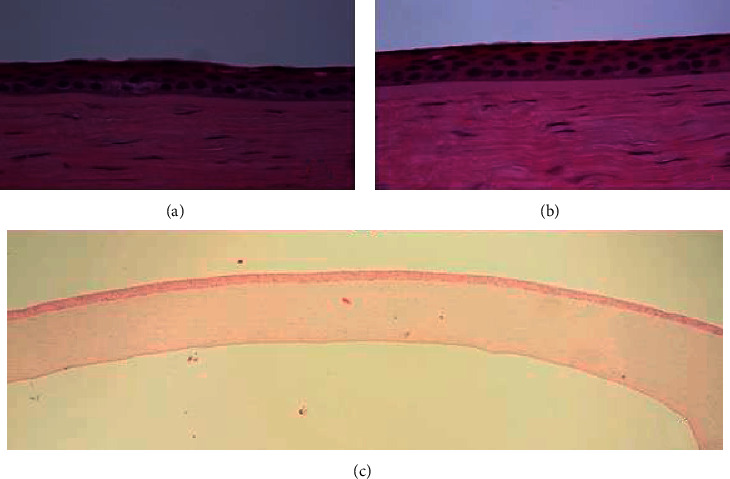
Pattern 3: central epithelium (a) as thick as peripheral epithelium (b). H&E-stained section. Original magnification 40x (a and b) and 5x (c). H&E: hematoxylin and eosin.

Three distinctive patterns of epithelial alteration of the central cornea were established. Pattern 1 was defined as central epithelium being focally thicker than the periphery (>10 *µ*m) (Figure 1); pattern 2 has the central epithelium thinner than periphery epithelium (>10 *µ*m) (Figure 2); and pattern 3 has central epithelium as thick as peripheral epithelium (±10 *µ*m) (Figure 3).

The commonest pattern was 3, accounting 40 of the 90 cases (44.4%), with similar thickness between central and peripheral epithelium.

The second commonest pattern was number 2, accounting for 35 of 90 cases (38.9%) showing marked reduced central epithelial thickness.

The most uncommon pattern was 1, accounting for 15 of 90 cases (16.7%).

The data of descriptive statistics of the sample are resumed in Tables 1–3.

There are statistically significant differences in all the patterns analyzed for both central and peripheral epithelium (*p* < 0.001).

There are statistically significant differences in the central corneal thickness between patterns 1 and 2 (*p* = 0.019) and patterns 1 and 3 (*p* = 0.009), but there are no significant differences in the central corneal thickness between patterns 2 and 3 (*p* = 0.735) and neither in the peripheral corneal thickness between all patterns (*p* = 0.342).

There are no statistically significant differences in all the patterns analyzed for both central (*p* = 0.08) and peripheral stroma (*p* = 0.32).

## 4. Discussion

It is very well-known that central corneal epithelium in KC responds with characteristic compensatory changes ranging from hypertrophy and hyperplasia to atrophy. However, we have not detected in the bibliographic search the description of the three patterns that we have found. These three distinctive histologic patterns could have a potentially diagnostic and prognostic value, and it would be very interesting to establish correlations with clinical parameters and/or with the use of therapeutic measures such as the use and time of contact lens wear. Unfortunately, this information is not available in the eye pathology laboratory, and it was not possible to obtain in this series except in a too small sample unable to make an appropriate inference analysis. Even so, the description of these epithelial response patterns has a definitive value, for example, when interpreting the clinical findings of the new imaging systems and for better planning of conservative treatments.

We found that epithelial thickness does not have correlation with stromal thickness neither in central nor peripheral zones, whose thickness is thinner than normal stroma in all patterns. In pattern 1, the total corneal thickness is higher in the central cornea. This data suggest that the corneal thickness is related to the epithelium in the mentioned pattern.

As mentioned, currently, the use of in vivo techniques such as ultrahigh resolution OCT have increased interest in the study of the corneal epithelial layer, and new therapeutic procedures have also been developed. However, the application of these innovations in the prognosis and treatment of KC has not been explored in detail [23–26]. Li et al. use Fourier-domain OCT to establish differences between normal corneal epithelium and corneal epithelium in subclinical KC corneas using pattern standard deviation variables, leading this to an early diagnosis of KC [27].

Other in vivo techniques such as high-resolution ultrasound digital scan could aid to diagnose early KCs that are classified as “normal” corneas using only topography techniques. Reinstein et al. described the compensatory changes of the epithelial layers overlapping stroma irregularities in early KC, acquiring a donut pattern, thinner over the cone and thicker in the surrounding area [28, 29].

Vega-Estrada et al. found a significant correlation between epithelial thickness measurements and the degree of visual impairment in keratoconus patients, using the anterior segment OCT (AS-OCT) with placido disc corneal topography and AS-OCT (MS-39; Costruzione Strumenti Oftalmici, Firenze, Italy). However, in this paper, the measurements were made in vivo compared with our study that was made in corneal buttons after the surgery [30].

Akcay et al. also described the compensatory role of the epithelial layer in KC. They evaluated the corneal topographical findings during CXL before and after removal of the corneal epithelium and found a significant increase in the value of the steepest keratometry and apical keratometry after removal of the epithelium, which means that this compensatory modification may mask the real value of corneal curvature and delay the diagnosis [31].

Proteomic studies had identified cell apoptosis, cell integrity alteration, and downregulation of miRNAs as the potential mechanisms for keratoconus epithelial degeneration. Using this knowledge, Wang et al. proposed impression cytology as a useful tool to collect and analyze the miRNA expression of corneal epithelia as a method for KC prediction [32].

Contact lens fitting as well as other treatments such as intracorneal ring segment implantations are highly dependent on the epithelial properties and response, with different degrees of treatment success and failure; thus, information on the existence of three different patterns may be useful for further analysis [33–35].

It is known that the prolonged use of gas permeable contact lenses may induce a reduction of corneal basal epithelial cell and stromal keratocyte density. Also, the use of scleral contact lenses may induce corneal changes such as corneal flattening [36, 37]. Due to these changes, it is possible that some of the patterns described are secondary to the modifications produced by the prolonged use of contact lenses; so, prospective studies with clinical data would be needed to corroborate it.

We like to emphasize that popularity and success of more conservative techniques such as cross-linking [38] or intrastromal corneal ring segments (approved by the FDA for the treatment of keratoconus in 2004) [39] are reducing the number of keratoplasties, and as mentioned, the widespread use of lamellar techniques is making increasingly rare to get full thickness corneal buttons. The latter are less prone to epithelial alteration due to the application of a microkeratome, the applanating lens of a femtosecond laser, or the manual dissection that are used for lamellar grafting.

The Department of Physics and Astronomy of the University of Waterloo is developing an ultrahigh resolution OCT, capable of obtaining accurate images of the corneal tissue. This in vitro and in vivo correlation would be the first established and could constitute a reference in a subsequent work aimed at the early diagnosis of this corneal ectasia, as well as assessing the effectiveness of certain treatments [40–42].

Future work is necessary to establish the clinical significance of these three morphological patterns by using ultrahigh resolution optical coherence tomography (UHR-OCT). Also, its potential use as predictive factors in the progression of KC needs to be elucidated.

## 5. Conclusions

Three distinctive histologic patterns that could potentially have a diagnostic and prognostic value in keratoconus patients were found. Further investigations are necessary to establish the clinical significance of these morphological patterns by using ultrahigh resolution optical coherence tomography.

## Figures and Tables

**Table 1:** Descriptive statistics of the sample (epithelial thickness).

		*N*	Mean	Standard deviation	Confidence interval 95%		Minimum	Maximum
					Inferior limit	Superior limit		
Central epithelial thickness	1	15	55.20	14.944	46.92	63.48	39	92
	2	35	27.89	8.362	25.01	30.76	10	50
	3	40	38.78	10.895	35.29	42.26	17	65
	Total	90	37.28	14.272	34.29	40.27	10	92

Peripheral epithelial thickness	1	15	34.27	9.573	28.97	39.57	20	50
	2	35	50.00	7.658	47.37	52.63	34	67
	3	40	42.13	9.635	39.04	45.21	22	60
	Total	90	43.88	10.449	41.69	46.07	20	67

**Table 2:** Descriptive statistics of the sample (total corneal thickness).

		*N*	Mean	Standard deviation	Confidence interval 95%		Minimum	Maximum
					Inferior limit	Superior limit		
Central corneal thickness	1	15	423.47	168.944	329.91	517.02	245	951
	2	35	339.69	96.681	306.47	372.90	136	581
	3	40	330.75	102.651	297.92	363.58	167	610
	Total	90	349.68	117.456	325.08	374.28	136	951

Peripheral corneal thickness	1	15	472.40	125.888	402.69	542.11	257	738
	2	35	494.63	118.345	453.98	535.28	256	698
	3	40	457.23	94.709	426.94	487.51	288	697
	Total	90	474.30	109.855	451.29	497.31	256	738

**Table 3:** Descriptive statistics of the sample (stromal thickness).

		*N*	Mean	Standard deviation	Confidence interval 95%		Minimum	Maximum
					Inferior limit	Superior limit		
Central stroma	1	15	368.27	163.881	277.51	459.02	187	870
	2	35	311.80	95.545	278.98	344.62	103	555
	3	40	291.98	102.050	259.34	324.61	102	568
	Total	90	312.40	113.971	288.53	336.27	102	870

Peripheral stroma	1	15	438.13	123.621	369.67	506.59	235	691
	2	35	444.63	115.502	404.95	484.30	206	640
	3	40	406.45	108.269	371.82	441.08	56	649
	Total	90	426.58	113.885	402.73	450.43	56	691

## Data Availability

The datasets used to support the findings of this study are available from the corresponding author upon reasonable request.
